# Preoperative factors predicting saphenous vein graft occlusion in coronary artery bypass grafting: a multivariate analysis

**DOI:** 10.1007/s00418-017-1574-4

**Published:** 2017-05-06

**Authors:** Agnieszka Malinska, Zuzanna Podemska, Bartlomiej Perek, Marek Jemielity, Piotr Buczkowski, Malgorzata Grzymislawska, Patrycja Sujka-Kordowska, Michal Nowicki

**Affiliations:** 1 Department of Histology and Embryology, Poznan University of Medical Sciences, Swiecickiego 6 St., 60-781 Poznan, Poland; 2 Department of Cardiac Surgery and Transplantology, Poznan University of Medical Sciences, Dluga 1/2 St., 60-101 Poznan, Poland; 3 Department of Anatomy, Poznan University of Medical Sciences, Swiecickiego 6 St., 60-781 Poznan, Poland

**Keywords:** CD133, Smooth muscle cells, Saphenous vein, Coronary artery bypass grafting, Prognosis

## Abstract

Saphenous vein segments are frequently used as aortocoronary bypass grafts, particularly in patients over 65 years of age. In the majority of patients, venous grafts maintain their patency for 5–6 years; however, some become occluded within 12 months after surgery. There are some defined predictive biological factors used to assess saphenous vein graft long-term patency rates, but little is known about molecular parameters for estimating the risk of early vein occlusion. The pathogenesis of this process involves the proliferation of stem cells, as well as progenitor cells, in the graft wall. Histologically, this is reflected by CD34 and CD133 expression in endothelial and smooth muscle cells. Thus, the aim of present work was to perform a multivariate analysis of stem cell and progenitor cell markers in saphenous vein graft walls before transplantation to arterial circulation and correlate these results with early graft occlusion. A total of 718 patients, who underwent coronary artery bypass grafting using a saphenous vein graft, were enrolled in this prospective study. CD34, CD133 and von Willebrand factor expression was evaluated via immunohistochemistry. A multivariate analysis revealed that strong CD133 expression in smooth muscle cells can be considered a risk factor for early graft failure. Our findings suggest that CD133 expression in smooth muscle cells of the tunica media in saphenous vein grafts obtained from coronary artery bypass graft patients before graft transplantation to coronary circulation might predict the possibility of early graft occlusion.

## Introduction

Coronary artery bypass grafting (CABG) is still one of the most common surgical procedures aimed at improving blood circulation in atherosclerotic coronary arteries (Kappetein et al. 2016). Donor blood vessels used in CABG are usually the internal thoracic artery (ITA) and great saphenous vein (SV) (Al-Sabti et al. 2013; Davierwala and Mohr 2015; Kappetein et al. 2016). The ITA is the vessel of choice in patients younger than 65 years (Davierwala and Mohr 2015). The SV is often used in older patients because it is significantly less likely to undergo reconstruction (arterialization) (Al-Sabti et al. 2013). In the majority of these patients, SV graft patency is maintained for 5–6 years (Harskamp et al. 2013). However, in approximately 10–15% of patients older than 65 years, SV grafts become occluded within 12 months after surgery (early occlusion) (Harskamp et al. 2013).

In general, graft patency is dependent on a number of factors, including the type and quality of graft used, the size of the coronary artery that the graft is anastomosed with, and, of course, the surgeon’s skill (Harskamp et al. 2013; Sarzaeem et al. 2010). The quality of a graft used in CABG refers to its specific histological architecture and/or the expression of certain tissue markers that might predict graft patency (Parolari et al. 2016). There are a number of well-known predictive factors used for assessing long-term ITA patency rates (Hickethier et al. 1999; Tomizawa et al. 1999). In the case of the SV, however, the number of indicators is more limited (Malinska et al. 2013; Perek et al. 2013). They include, to some extent, the expression of caveolin-1 and caveolin-3 in SV smooth muscle cells (SMCs) and the macrophage concentration in the vein wall (Malinska et al. 2013; Perek et al. 2012). These parameters can be useful for estimating the risk of vein occlusion (when transplanted to coronary circulation) over periods exceeding 12 months (Perek et al. 2012). At present, it is not possible to accurately calculate the risk of earlier SV occlusion after CABG.

The pathogenesis of early SV graft occlusion after transplantation to arterial circulation follows the proliferation of stem cells and progenitor cells in the graft wall (Timmermans et al. 2009). Histologically, the presence of these cell populations is reflected by CD34 and CD133 expression in the endothelium and SMCs (Busch et al. 2015; Irollo and Pirozzi 2013). A number of studies have evaluated the morphological characteristics of already-occluded explanted SV grafts and cite evidence of CD34+ and CD133+ cell involvement in neointimal formation (Busch et al. 2015; Shoji et al. 2014). There are, however, no studies examining whether the higher expression of CD34 and CD133 in SV grafts prior to their transplantation is a suitable marker for increasing proliferative capacity of cells in the graft after CABG, or not.

To address this issue, the aim of the present work was to perform a multivariate analysis of stem cell and progenitor cell markers in SV graft walls before transplantation to arterial circulation and determine if the results correlate with early graft occlusion.

## Patients and methods

### Study group

The Poznan University of Medical Sciences Local Ethics Committee approved the research protocol (No. 1201/08), and informed written consent was obtained from all participants. In total, 718 consecutive patients [481 males (67%) and 237 females (33%)], who underwent elective on-pump CABG with the use of at least one venous aortocoronary bypass graft between September 2008 and October 2015, were enrolled in this prospective study. Two surgeons implanted 1568 SV grafts in this group of patients.

Patients who needed emergent surgery and individuals diagnosed with any peripheral vein pathologies were excluded from the study. The other exclusion criteria included a class IV NYHA classification, cardiogenic shock, *arteriosclerosis obliterans*, diabetes, chronic renal failure and chronic renal disease. For all study participants the routine biochemical blood analysis were performed (COBAS INTEGRA 800 Analyzer Roche, Switzerland) including triglyceride levels, cholesterol fraction plasma concentrations (VLDL, LDL, HDL) and lipoprotein A levels. Basic preoperative demographics and clinical history data are summarized in Table 1.

**Table 1:** Preoperative patient characteristics

	Value
Age (years)	61.8 ± 5.3; range 52–85 years
Gender (male/female)	481/237
Hyperlipidemia (%)	57.5
Smoking (%)	36.3
Lipoprotein A (mg/dL)	31.8 ± 6.7
Family history of ischemic heart disease (%)	34.9
Number of grafts anastomosed in each patient	2.7 ± 1.2

### Surgical procedure and sample collection

All surgeries were performed via a median sternotomy, and the patients underwent a conventional CABG with extracorporeal circulation and moderate hypothermia (on-pump CABG). SV grafts were obtained through a full-length thigh incision made above the path of the vein (Timmermans et al. 2009). Key points of the procedure included minimal manipulation of the graft (“no-touch” technique), avoiding extensive dilation of the conduits, using low-intensity electrocautery and controlling the branches with stainless-steel vascular clips. In all cases, the distal part of the harvested SV segment (at least 15–20 mm in length) was saved for subsequent laboratory studies.

### Follow-up

Twelve months after CABG, all the patients were evaluated via 64-slice multidetector computed tomography (CT). SV grafts with no evidence of luminal stenosis were classified as “patent”. Diseased conduits were classified as “single” when there was a single, local lesion; “multiple” when there was more than one lesion but it was still not totally occluded; and “occluded” when no lumen was observed in the CT examination.

### Immunohistochemistry

Briefly, the venous segments were carefully rinsed in 0.9% NaCl using a VASOSHIELD Pressure Controling Syringe (Maquet Getinge Group, Rastatt, Germany) and fixed in Bouin’s solution for 24 h. They were then dehydrated and embedded in paraffin blocks using a routine procedure. Finally, they were cut into 3–4 µm sections on a semi-automatic rotary microtome (Leica RM 2145, Leica Microsystems, Nussloch, Germany). Each SV sample (taken from separate patients) was cut into approximately 30 paraffin sections. For the immunohistochemical (IHC) analysis of each protein expression, we used 27 sections (nine for CD34, nine for CD133 and nine for vWF). One slide (with 3 saphenous vein sections) was dedicated for negative control. All of IHC analyses involved the StreptABComplex/HRP method, modified by using biotinylated tyramide (Dako Catalyzed Signal Amplification System, Peroxidase, K1500, DakoCytomation A/S, Glostrup, Denmark) (Warford et al. 2014). After deparaffinization with xylene and gradual rehydration, endogenous peroxidase activity was blocked with 10% hydrogen peroxide (v/v) for 10 min. In consecutive stages of the IHC analysis, the indirect ABC technique was performed, with the following steps: (1) preincubation with the appropriate normal goat serum in phosphate-buffered saline (PBS) for 30 min at room temperature, (2) incubation with the first specific antibody overnight at 4 °C in a hybridization chamber, (3) incubation with the secondary antibody for 60 min at room temperature (4) final antigen–antibody complex staining using 0.5% 3, 3′-diaminobenzidine (DAB; Sigma Chemical Co., St. Louis, MO, USA).

The following monoclonal mouse anti-human antibodies were used: anti-CD34 (Dako; M7165; diluted 1:200), anti-CD133 (Novus Biologicals Europe, Cambridge, UK; NB120-16,518; diluted 1:300) and anti-von Willebrand factor (vWF; Dako; M0616; diluted 1:100). The last antibody was used to identify advanced-stage endothelial cell (EC) maturation. After IHC staining, tissue sections were counterstained with hematoxylin for 30 s to 5 min depending on the expected intensity of the IHC staining. The intensity of SMCs protein expression was assessed using the semi-quantitative IRS (immuno-reactive score) scale described by Remmele and Stenger (Remmele and Schicketanz 1993). Based on this scale, protein expression was defined as negative (IRS 0–1), positive and weak (IRS 2–3), positive and moderate (IRS 4–6) or strong (IRS 8–12) (Table 2). Quantitative assessments of CD34, CD133 and vWF expression in the ECs of the tunica intima and in the SMCs of the tunica media were performed by comparing the length of the immunopositive endothelium to the total length of the endothelium. The ratio of positive ECs was calculated with the use of AxioVision image processing software (Carl Zeiss MicroImaging GmbH, Göttingen, Germany) (Table 2). All tissue sections were analyzed under an AxioImager Z.1 light microscope, and selected pictures were captured with the attached AxioCam MRc5 digital camera (Carl Zeiss).

**Table 2:** Correlation between saphenous vein graft immunohistochemical characteristics, the target vessels and SV graft patency 1 year after surgery

Variable	Categories	%	Patent	Single	Multiple	Occluded
CD34	Positive intimal ECs ≥90%	93.2	92.6*	1.2	3.3	2.9
	Positive luminal ECs 80–90%	5.5	91.1*	2.4	3.7	2.8
	Positive intimal ECs <80%	1.3	2.2	4.6	4.9	88.3*
CD133	Positive intimal ECs ≥90%	94.1	83.3*	6.7	5.9	4.1
	Positive intimal ECs 80–90%	3.3	82.1*	6.6	5.4	5.9
	Positive intimal ECs <80%	2.6	83.7*	5.5	3.8	7.0
	Positive SMCs ≥8 IRS	11.2	17.9	23.2	18.2	40.7**
	Positive SMCs 4–6 IRS	28.2	34.5***	19.2	21.1	25.2
	Positive SMCs ≤3 IRS	70.6	89.9*	2.4	2.9	4.8
vWF	Positive intimal ECs ≥90%	89.9	84.1*	3.9	4.1	7.9
	Positive intimal ECs 80–90%	5.7	82.2*	4.1	4.0	9.7
	Positive intimal ECs <80%	4.4	83.1*	3.9	3.8	9.2
Target vessel	D	34.6	89.8*	2.9	3.1	4.2
	LAD	5.5	74.6*	9.9	5.3	10.2
	LCx	17.8	91.3*	3.2	2.8	2.7
	LM	19.2	92.0*	1.8	1.9	4.3
	PDA	11.0	88.8*	5.6	4.1	1.5
	RCA	11.9	76.1*	9.9	4.5	9.5

*SV* great saphenous vein, *Patent* SV graft normally filled with contrast, *Single* localized stenosis in SV graft, *Multiple* multiple stenoses in SV graft, *Occluded* no contrast in SV graft, *ECs* endothelial cells, *SMCs* smooth muscle cells, *IRS* immuno-reactive score, *vWF* von Willebrand factor, *D* diagonal coronary artery, *LAD* left anterior descending coronary artery, *LCx* left circumflex coronary artery, *LM* left marginal coronary artery, *PDA* posterior descending coronary artery, *RCA* right coronary artery

* *p* < 0.0001 for evaluated SV graft characteristics in relation to its patency after 1 year

** *p* = 0.008 for strong CD133 expression in SMCs of SV graft in relation to its critical occlusion within 1 year since CABG

*** *p* = 0.048 for moderate CD133 expression in SMCs of SV graft in relation to its patency after 1 year

IHC analyses of protein expression in each vessel section were performed within ten representative microscopic fields (×200 magnification). All of the analyses were evaluated independently by two scientists on coded samples and included positive and negative controls. The negative controls consisted of specimens incubated with non-immune IgG1 (X-0931, Dako) and sections for which the primary or secondary antibody had been omitted. All of the sections from a vein sample of an individual patient were processed in the same IHC experiment. In addition, to determine the consistency of CD34, CD133 and vWF reactivity, serial sections obtained from the human tonsil, kidney and skin, respectively, were used as external positive controls. Palatine tonsils were obtained from the patients who had undergone tonsillectomy for idiopathic tonsillar hyperplasia, control kidney was available from a patient diagnosed with stage I clear cell papillary renal cell carcinoma (a control tissue was obtained from an unaffected area), skin tissue was derived from the healthy control participants involved in our previous study regarding neuropathy in type 1 diabetes. All the tissues came from the archive of the Department of Histology and Embryology, Poznan University of Medical Sciences.

### Statistical analysis

The results were statistically reviewed every 3 months to enable the study to be terminated early if clear results emerged. Four interim analyses were performed during the trial. However, the results were not statistically significant until the final analysis was performed (12 months after the surgery). The interim analyses have not been considered in the sample-size calculation.

Continuous or interval-related variables are expressed as the mean ± SD. The statistical analysis was performed in the following steps: (1) A Chi-squared test was used to define the association between early SV occlusion and the categorical variables; (2) a one-way analysis of variance (one-way ANOVA) was employed to evaluate the association between SV graft patency and the continuous variables; (3) Bonferroni corrections were used to adjust alpha for all of the multiple comparisons performed in the study; and (4) finally, a logistic regression model was constructed to calculate the odds ratios for the variables that might have influenced graft patency. A value of *p* < 0.05 was considered to be statistically significant.

All of the statistical analyses were performed using the Statistica 12.0 PL software package (StatSoft, Poland).

## Results

One year after CABG, 1290 SV grafts (81.3%) were classified as “patent”. One or more non-occlusive lesions were found in 134 conduits (8.5%). Finally, complete occlusion developed in 162 SV grafts (10.2%). The mean age of patients who did not developed any lesions within SV grafts was 60.8 ± 4.9 years (range from 52 to 79 years). Study participants who developed a non-critical SV graft occlusion ranged in age from 58 to 83 years (mean 62.5 ± 5.5 years). Finally, in 72 patients, no lumen was observed in the SV graft (range from 59 to 85 years; mean age 64.1 ± 5.2 years). Differences in the mean ages of the study patients were not significant.

### Immunohistochemistry

**Fig. 1:**
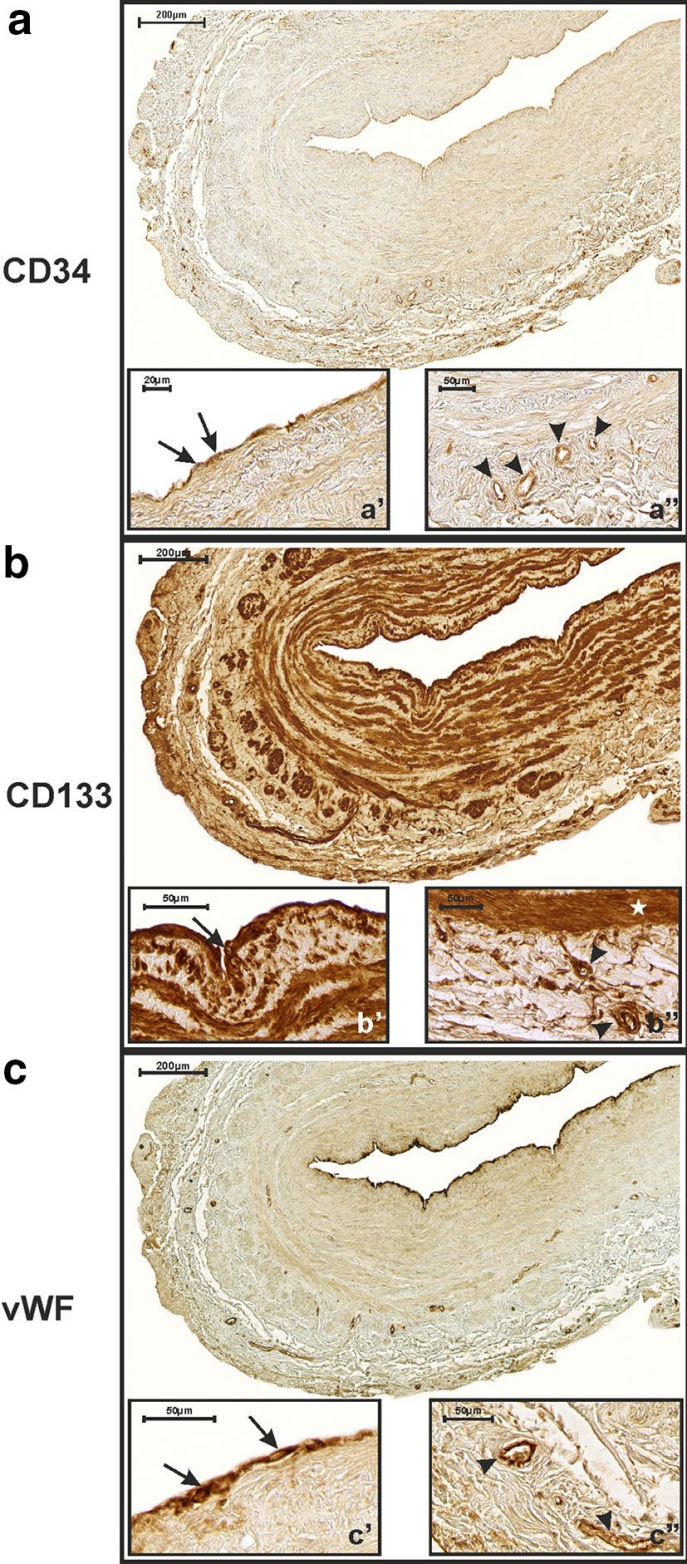
Immunohistochemical detection of CD34 (**a**), CD133 (**b**) and vWF (**c**) expression in a saphenous vein graft obtained from a 59-year-old CABG patient who developed a complete graft occlusion 11 months after surgery. CD34 is expressed within endothelial cells present in the tunica intima (**a′** *arrows*) of the studied graft, as well as in small blood vessels and capillaries present in the tunica adventitia (**a″** *arrow heads*). CD133 is strongly expressed in intimal endothelial cells (**b′** *arrow*) and smooth muscle cells (**b″** *asterisk*) (IRS = 12). Small adventitial blood vessels and capillaries are also CD133-positive (**b″** *arrow heads*). Finally, vWF factor immunoreactivity is observed within intimal endothelial cells (**c′** *arrows*). Only a very few small blood vessels, as are present in the adventitia, are vWF-positive (**c″** *arrow heads*)

**Fig. 2:**
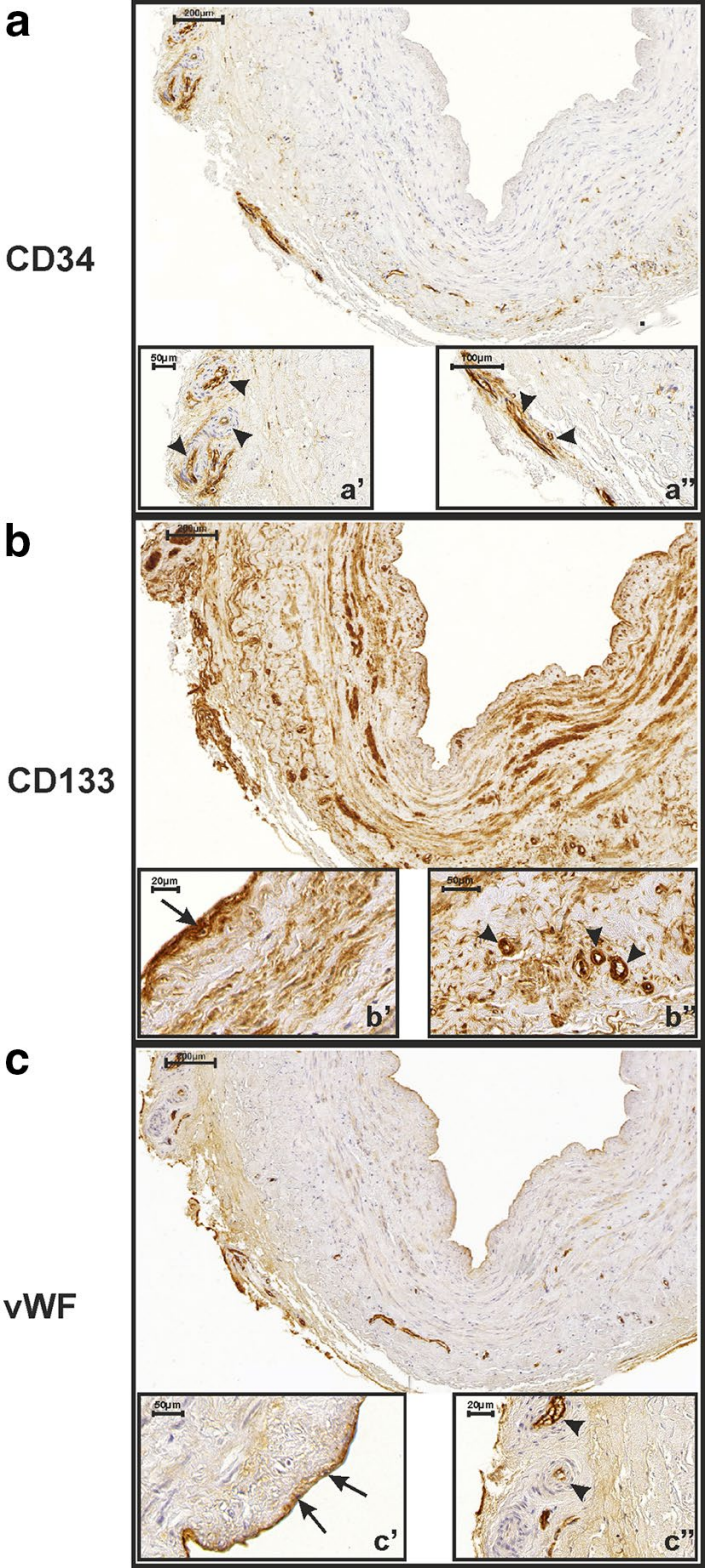
CD34 (**a**), CD133 (**b**) and vWF (**c**) immunoreactivity in a saphenous vein graft obtained from a 72-year-old CABG study participant who developed an early graft occlusion (9 months after CABG procedure). CD34 is not expressed in intimal endothelial cells. It is only present in some of the small blood vessels present in the adventitia (**a′**, **a″** *arrows heads*). CD133 is strongly expressed in smooth muscle cells (IRS = 8), as well as in the endothelium lining the graft lumen (**b′** *arrow*) and vasa vasorum (**b″** *arrow heads*). vWF is expressed in intimal endothelial cells (**c′** *arrows*) and individual capillaries present in the adventitia (**c″** *arrow heads*)

**Fig. 3:**
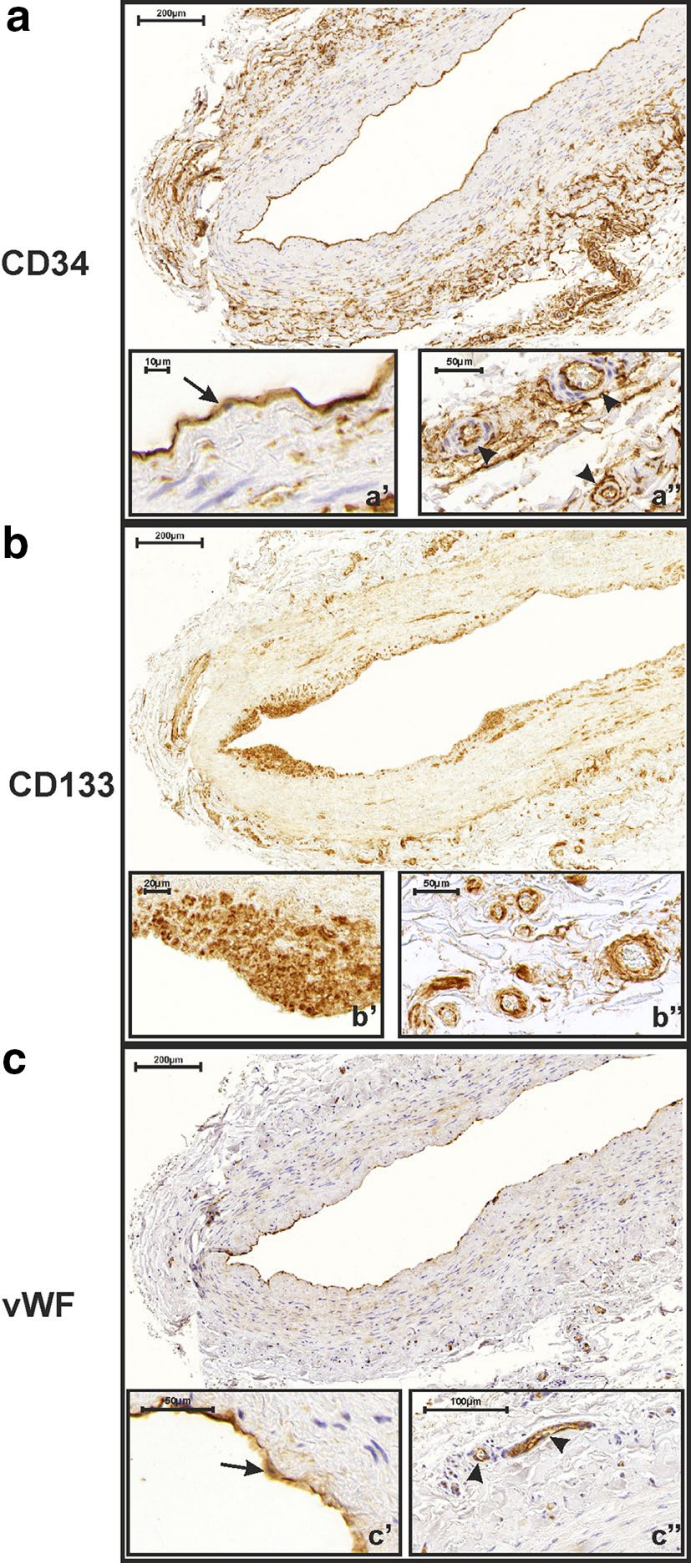
Immunohistochemical detection of CD34 (**a**), CD133 (**b**) and vWF (**c**) expression in a 73-year-old patient who did not develop saphenous vein graft occlusion. CD34 is strongly expressed in the intimal endothelium of the saphenous vein graft (**a′** *arrow*). There are no immuno-negative endothelial cells in this area. Moreover, graft capillaries present in the adventitia are also CD34 positive (**a″** *arrow heads*). Notice the weak CD133 expression in smooth muscle cells of the tunica media. Numerous capillaries are CD133 positive (but CD34 negative) in the tunica intima (**b′**). Individual capillaries in the adventitia are also CD133-positive (**b″**). VWF is expressed in intimal endothelial cells (**c′** *arrow*) and individual capillaries present in the adventitia (**c″** *arrow heads*)

CD34 was expressed in the ECs present in the tunica intima of SV grafts and in individual capillaries located in the walls of grafts. In more than 93% of anastomosed grafts, the percentage of CD34+ intimal ECs was ≥90 (Fig. 3a, a′). Only 5.5% of the studied grafts showed CD34 expression in ECs between 80 and 89.9% (Fig. 1a, a′), and 20 grafts (1.28%) showed CD34 expression of <80% (Fig. 2a). The mean percentage of CD34 immunopositive cells lining the tunica intima of SV grafts was 94.2 ± 4.9.

Immunohistochemistry involving the use of specific anti-CD133 antibodies revealed strong CD133 expression (IRS ≥ 8) in the SMCs of the tunica media in 178 SV grafts (11%) (Fig. 1b, b″). Moderate expression was estimated in 447 grafts (28%) (Fig. 2b). Weak or no CD133 expression (IRS ≤ 3) was found in 961 SV grafts (61%) (Fig. 3b). CD133 was also expressed in the ECs forming the tunica intima and lining the *vasa vasorum* of the adventitia in all study participants. The mean percentage of immunopositive ECs in the tunica intima was 92.2 ± 3.6 (Figs. 1b, b′, 2b, b′, 3b).

Finally, vWF was expressed in all of the studied SV grafts. It was present in intimal ECs and in individual capillaries located in the adventitia (Figs. 1c, c′, 2c, c′, 3c, c′). The mean ratio of vWF-positive ECs in the tunica media was 93.1 ± 3.2.

### Predictive factors

Early critical SV occlusion did not correlate with triglyceride levels, cholesterol fraction plasma concentrations (VLDL, LDL, HDL), or lipoprotein A levels. What is more, CABG subjects who required a subsequent surgery had no diagnosed active metabolic diseases and did not have decompensated arterial hypertension (Table 1).

Table 2 summarizes the correlation between SV graft IHC characteristics, the target vessels, and SV patency after 1 year. Almost all evaluated variables concerning the SV graft IHC status had a significant correlation with graft patency (*p* < 0.0001). The only exception was ^1^strong CD133 expression in the SMCs of SV graft (IRS ≥ 8) and ^2^CD34+ expression in the ECs < 80%, which correlated with critical graft occlusion within 12 months after CABG. The analysis was performed using a Chi-squared test. The distribution of all variables was evaluated, and *p* values refer to the difference in the frequency distribution of all the groups.

In the next step, a logistic regression model was constructed to analyze the SV grafts that were found to be “occluded” on CT imaging. In the multivariate analysis, only strong CD133 expression in the SMCs had a significant odds ratio (Table 3).

**Table 3:** Odds ratio of the variables upon univariate and multivariate analyses for occluded saphenous vein grafts

Characteristic	Univariate analysis			Multivariate analysis		
	*B*	OR	*p* value	*B*	OR	*p* value
Age	0	1.00	0.812	0.01	1.01	0.766
Male	−0.05	0.95	0.844	0.84	2.31	0.069
Hyperlipidemia (%)	0.49	1.67	0.075	0.08	1.11	0.743
Smoking (%)	0.45	1.61	0.095	0.32	1.56	0.102
Lipoprotein A (mg/dL)	0.23	1.37	0.236	0.42	1.08	0.788
Family history of ischemic heart disease (%)	−0.44	0.66	0.086	0.94	2.54	0.134
CD34+ intimal ECs ≥90%	0.11	1.08	0.655	0.36	1.58	0.098
CD34+ intimal ECs 80–90%	0.44	1.61	0.096	0.77	1.98	0.086
CD34+ intimal ECs <80%	0.55	1.88	0.022	1.09	2.88	0.066
CD133+ intimal ECs ≥90%	0.12	1.10	0.639	0.38	1.61	0.102
CD133+ intimal ECs 80–90%	0.32	1.44	0.238	0.22	1.22	0.544
CD133+ intimal ECs <80%	0.21	1.22	0.445	1.10	2.90	0.064
CD133+ SMCs ≥8 IRS	1.56	4.88	0	1.82	8.08	0
CD133+ SMCs 4–6 IRS	0.42	1.58	0.072	1.22	2.98	0.061
CD133+ SMCs ≤3 IRS	0	1.00	0.811	0.01	1.01	0.755
vWF+ intimal ECs ≥90%	0.14	1.12	0.611	0.42	1.82	0.088
vWF+ intimal ECs 80–90%	0.30	1.42	0.286	0.20	1.20	0.642
vWF+ intimal ECs <80%	045	156	0.064	0.88	1.90	0.264

*B* regression coefficient, *OR* odds ratio, *ECs* endothelial cells, *SMCs* smooth muscle cells, *IRS* immuno-reactive score, *vWF* von Willebrand factor

## Discussion

The basic observation from the present study is the potent, predictive role of CD133 (present in the SMCs of the tunica media in SV grafts) in early graft occlusion in CABG patients. Generally, the risk of SV graft occlusion in CABG is significantly higher in younger patients (Harskamp et al. 2013). For this reason, CABG procedures involve the use of the internal thoracic artery in this group of patients. In older patients, the SV seems to be the vessel of choice for bypass grafting. Our studies indicate that early SV graft occlusion in CABG patients was independent of their age. The only marker that predicted the risk of critical graft occlusion was CD133 expression in the SMCs in SV graft transplants at the moment of the primary CABG procedure. In our opinion, patients with this risk factor should be allocated to the high-risk group for graft occlusion and would benefit from planned multidetector CT examinations performed at least once within 12 months after CABG.

Problems related to the postoperative follow-up of CABG patients are still unresolved. Some studies suggest that the lack of CD34 expression in ECs of the tunica intima of SV transplants might indicate a higher risk of early graft occlusion (Viaro et al. 2007). Another report considered the presence of immune cells (including macrophages) in the walls of transplanted vessels (Malinska et al. 2013). These observations, however, were made on much younger patients compared with those in the present report.

Regardless of the above-mentioned studies, SV grafts remain patent as long as the process of graft reconstruction (arterialization) does not occur (Berard et al. 2013; Perek et al. 2012). Briefly, SV arterialization follows SMC proliferation, leading to medial hypertrophy (Angelini and Jeremy 2002). An essential element of these alterations is the process of SMC dedifferentiation indicated by, among other events, CD133 expression (Brzoska et al. 2011). Such cells also acquire the ability to migrate into the tunica intima, resulting in neointimal formation (Angelini and Jeremy 2002; Brzoska et al. 2011).

Repetitive endothelial injury is also believed to induce neointimal hyperplasia in an animal model (Xiang et al. 2014). In contrast, early lesions by neointimal hyperplasia in humans begin at sites with morphologically intact endothelium (Hillebrands et al. 2005; Little et al. 2008). Graft occlusion following CABG in humans is a complex process and includes an increase in endothelial permeability, enhanced leukocyte adhesion, SMC dedifferentiation and alterations in the expression of EC gene products (Khaleel et al. 2012).

It must be emphasized that in our study, CD133 expression in SV grafts was estimated before transplantation into high-pressure circulation. This means that CD133 might indicate also a cell’s potency for rejuvenation. We can suppose that a strong CD133 expression is a predictor for “healing response” that begins before graft transplantation. However, such a hypothesis needs a further study.

Translation of basic knowledge to clinical trials has recently introduced a novel therapeutic application of autologous CD133-positive endothelial progenitor cells for the adjuvant treatment of acute and chronic myocardial ischemia (Bongiovanni et al. 2014; Nagamine et al. 2004). In line with the studies presented above, such a treatment should be carefully evaluated before being implemented in patients who have previously undergone CABG with the use of a SV graft. An increase in the number of circulating CD133+ cells in these patients, which possess the ability to migrate into the blood vessel wall, might decrease the longtime functionality of vein transplants in coronary circulation.

We conclude that CD133 expression in the SMCs of the tunica media of SV grafts obtained from CABG patients before transplantation into coronary circulation might predict the possibility of early graft occlusion. CD34 and vWF are useful markers for the evaluation of the ECs in SV grafts; however, they do not have prognostic significance.
